# Shedding light on the black box models of the cell

**DOI:** 10.1111/1751-7915.12489

**Published:** 2017-01-03

**Authors:** Jose Ignacio Jiménez

**Affiliations:** ^1^ Faculty of Health and Medical SciencesUniversity of SurreyGuildfordGU2 7XHUK

The popularity of mathematical models applied in biological systems has increased exponentially since the early 2000s with the advent of fields like Systems and Synthetic Biology. In this perspective, I would like to focus on a particular kind of model used to explain dynamic behaviours of biological systems. In this case, we use models to explain the changes in the concentration of elements over time, regardless of the ‘size’ of these elements. The same equations that are used to explain macroscopic changes in the ecology of a niche (species, cells, viruses) may also be used to explain the dynamics of molecular components of the cell (proteins, transcription factors, mRNAs, etc.). These models capture in just a few elegant equations the macroscopic behaviour of a biological system and have had an undeniable impact in biology (Jungck, 1997). To cite a couple of famous examples, we now refer to Lotka–Volterra or Turing models to explain, respectively, oscillatory processes in molecular biology and developmental mechanisms of cells and organs.

All these models share a common property. They use a mathematical abstraction to explain empirical observations and data that were made prior to the development of the model. In other words, the relevant biological data exist before the model is built. Initial versions of the model are based on these data, and subsequently, further experiments can be designed to refine the model structure and identify relevant parameters to make the abstraction as realistic as possible. Such models are extremely useful and can help to identify new properties or elements of biological systems.

What is considered the first mathematical model in biology falls into a different category. In the year 1202, long before calculus was invented, the mathematician Fibonacci tried to predict how many rabbits could breed over time in ideal circumstances starting from a pair of female–male rabbits. He assumed that the animals would never die and that females could only give birth to a new pair of female and male rabbits each time. This is clearly an oversimplified model that is far from biological reality. It renders a series of numbers – the celebrated ‘Fibonacci numbers’ – that are very different from the numbers of animals that would have been determined empirically. The discrepancy between prediction and observation in this case highlights the difficulty of building models with predictive power prior to having appropriate empirical evidence. Engineers often use models to explore the ‘design space’ of new products before actually building them, but are generally working with systems assembled from well‐defined components. In the context of Synthetic Biology, which has among its goals the standardized design and construction of novel dynamic systems, such as genetic circuits, the underlying components (‘parts’) are often quite poorly characterized, and this leads to a corresponding decrease in the predictive power of our models.

Synthetic biologists have managed to address the problem of the discrepancies between model predictions and experimental observations by following the so‐called engineering cycle. This consists of employing successive iterations of design, modelling, construction and testing of novel biological systems. Although this iterative cycle has proven to be a useful tool, it is time and resource intensive. Thanks to advances in DNA synthesis and screening, it is now possible to automate the design and construction of certain genetic circuits (Nielsen *et al*., 2016). This method shows great promise and allows significant reductions in the time required to obtain working constructions. It requires, however, the use of well‐characterized parts that may not be suitable for all applications.

Besides automation, another possibility to facilitate the design of genetic circuits is to improve the way we build models. The question that remains open and that many people are trying to answer is why are models inaccurate? I have posed this question to different audiences showing for illustration the side‐by‐side comparison of the theoretical and experimental behaviour of a synthetic oscillator. The model predicts elegant and periodical oscillations, whereas the results obtained in the laboratory show minimal damped oscillations that are attenuated over time. Despite the fact that the model captures, at least qualitatively, the behaviour of the system, in most cases, the audience agrees that the discrepancy is too high to consider the model to be acceptable as a design tool. I would argue that in this particular case, and in many others, the difference between prediction and observation results from the poor assumptions on which the model is based. Unfortunately, these assumptions are rather common in the context of genetic circuits: the models are normally built by considering the interactions between the components of the circuit while neglecting the potential interplay with the cell containing the circuit. Essentially, the cell is considered as a black box, a mere vessel wherein the circuit is contained.

Most mathematical biologists would agree that a model is only as good as the assumptions on which it is based. Some, like the mathematician Jeremy Gunawardena (Harvard Medical School), would even go further and argue that models are ‘accurate descriptions of our pathetic thinking’ (Gunawardena, 2014). So, how do we improve models of genetic circuits? The answer is obvious: by making better assumptions, starting with the fact that the cell itself is not a black box but rather, a complex economy in which scarce resources are carefully invested depending on fluctuating environmental conditions. This means that synthetic genetic circuits do not exist in isolation but in competition with the rest of the genes of the cell. Therefore, to increase the predictive power of models, we would need to shed light on the black box, by building a model of the whole cell. Given the high number of chemical species inside a cell, obtaining a dynamical model that explains how the concentrations of all of them change over time is far from a trivial problem. Progress is being made in the global understanding of bacteria with small genomes composed of just a hundred genes. Unfortunately, this kind of approach is beyond our current computational capabilities when considering commonly used bacteria, such as *Escherichia coli*, which contains around 4,400 genes. The solution is to use the appropriate level of abstraction. For example, recent studies have come up with imaginative ways of building global models that do not consider all genes and proteins as individual species but rather model the cell behaviour as the result of trade‐offs between limited amounts of three elements: energy (i.e. ATP and NADPH), proteins and free ribosomes (Weiße *et al*., 2015). By incorporating previous phenomenological evidence, this model is capable of capturing the interplay between growth rate and the amount of ribosomes, as well as predicting some host–circuit interactions. Although this model may be seen as an oversimplification of the physiology of the cell, it provides us with a valuable framework that over time can be populated with more accurate descriptions of other relevant cellular components that can describe more complex interplays between genetic circuits and the host.

This brings us to the following question: What key additional features of the cell should be incorporated in current models while retaining a tractable level of complexity? There are some, despite the limitations. Metabolic networks, for a start, can be described using flux balance analysis and have been successfully merged with regulatory networks (Covert *et al*., 2004). Additionally, global allocation of transcriptional and translational resources could be implemented with little computational cost (Lerman *et al*., 2012). Finally, it is now becoming widely accepted that bacterial cells are not mere containers of homogenously distributed chemical species. Instead, recent evidence indicates that the cell cytoplasm has a structure and the different partitions in which it is organized have an effect on the way genes are expressed (Castellana *et al*., 2016). Accounting for the physical location of genetic circuits, competing host genes, and cell resources, is necessary to understand unexpected couplings in protein synthesis.

All these properties would help to improve our current models in such a way that that they remain computationally tractable, while being based on a more realistic set of assumptions. This would greatly improve the accuracy of their predictions, increase their usefulness as design tools and contribute to making biology a more engineering‐friendly discipline.
